# Roles of vaginal flora in human papillomavirus infection, virus persistence and clearance

**DOI:** 10.3389/fcimb.2022.1036869

**Published:** 2023-01-04

**Authors:** Mi Zeng, Xin Li, Xiaoyang Jiao, Xiaochun Cai, Fen Yao, Shaomin Xu, Xiaoshan Huang, Qiaoxin Zhang, Jianqiang Chen

**Affiliations:** ^1^ Department of Cell Biology and Genetics, Shantou University Medical College, Shantou, Guangdong, China; ^2^ Guangdong Provincial Key Laboratory of Infectious Diseases and Molecular Immunopathology, Shantou, Guangdong, China; ^3^ Department of Gynecology and Obstetrics, Chenghai District People’s Hospital, Shantou, Guangdong, China; ^4^ Department of Pharmacology, Shantou University Medical College, Shantou, Guangdong, China; ^5^ Longhu Hospital, The First Affiliated Hospital of Shantou University Medical College, Shantou, China; ^6^ The First Affiliated Hospital of Shantou University Medical College, Shantou, China

**Keywords:** human papillomavirus, vaginal microbiota, lactobacillus, 16S ribosomal DNA sequencing, probiotics

## Abstract

Vaginal flora plays a vital role in human papillomavirus (HPV) infection and progression to cancer. To reveal a role of the vaginal flora in HPV persistence and clearance, 90 patients with HPV infection and 45 healthy individuals were enrolled in this study and their vaginal flora were analyzed. Women with HPV infection were treated with *Lactobacillus* in the vaginal environment as a supplement to interferon therapy. Our results indicated that patients with high risk HPV (Hr-HPV) 16/18 infection had a significantly higher alpha diversity compared with the healthy control (*p* < 0.01), while there was no significant difference between the non-Hr-HPV16/18 group and the controls (*p* > 0.05). Patients with multiple HPV infection had insignificantly higher alpha diversity compared with single HPV infection (*p* > 0.05). The vaginal flora of patients with HPV infection exhibited different compositions when compared to the healthy controls. The dominant bacteria with the highest prevalence in HPV-positive group were *Lactobacillus iners* (n = 49, 54.44%), and the top 3 dominant bacteria in the HPV-persistent group were *Lactobacillus iners* (n = 34, 53.13%), *Sneathia amnii* (n = 9, 14.06%), and *Lactobacillus delbrueckii* (n = 3, 4.69%). Patients with HPV clearance had significantly lower alpha diversity, and the flora pattern was also different between groups displaying HPV clearance vs. persistence. The patients with persistent HPV infection had significantly higher levels of Bacteroidaceae, Erysipelotrichaceae, Helicobacteraceae, Neisseriaceae, Streptococcaceae (family level), and *Fusobacterium*, *Bacteroides*, *Neisseria*, and *Helicobacter* (genus level) than patients who had cleared HPV (*p* < 0.05).

**Importance:**

Our study revealed differences in vaginal flora patterns are associated with HPV persistence and its clearance. Interferon plus probiotics can greatly improve virus clearance in some patients. Distinguishing bacterial features associated with HPV clearance in patients would be helpful for early intervention and reverse persistent infection.

## Introduction

Human papillomavirus (HPV) infection is one of the most common sexually transmitted infections, and is also the leading cause of cervical cancers (Lehtinen et al., 2019). Up to now, 396 distinct HPV subtypes have been reported (Bzhalava et al., 2014). Genital HPVs can be subdivided into high- and low-risk types, with 13 being identified as high-risk HPVs (Hr-HPVs) (Walboomers et al., 1999), and the two most common cervical Hr-HPVs are HPV16 and 18. Most low-risk types of HPV infections resolve over time. However, persistent cervical Hr-HPV infections play a crucial role in the development of cervical cancer (Schiffman et al., 2016). HPV infection is actively involved in cervical epithelial transformation (Wilkinson et al., 2015; Curty et al., 2017). Although approximately 70% of cervical cancer cases worldwide are caused by HR-HPV (Clifford et al., 2003; Oliveira and Schirger, 2003; Du et al., 2011; Chan et al., 2019), not all people with HPV infection actually end up developing cancer and only a small percentage of Hr-HPV infections develop into cervical cancer, indicating that virus infection is not sufficient for cancer development, additional factors may involve in HPV inducing cervical cancer (Usyk et al., 2020).

Recent studies have shown factors, including integrity of epithelial surface, mucosal secretions, immune regulation, and the local microbiota, play a part in the development of HPV infection to cancer (Pyeon et al., 2009; Fernandes et al., 2015; Schiffman et al., 2016). More than 200 bacterial species comprise the vaginal flora of healthy women, which are mainly dominated by one of the four most prevalent *Lactobacillus* species: *Lactobacillus crispatus* (*L. crispatus*)*, Lactobacillus iners* (*L. iners*), *Lactobacillus gasseri* (*L. gasseri*), and *Lactobacillus jensenii* (*L. jensenii*). *Lactobacillus* spp. form barriers against colonization of bacterial vaginosis (BV)-associated bacteria by maintaining a low pH (Mastromarino et al., 2014; Breshears et al., 2015). It is essential for maintaining cervical epithelial barrier function which inhibits infection of basal keratinocytes by HPV (Borgdorff et al., 2016). BV is also connected with an increase in the production of epithelial lining-degrading enzymes that can allow the initiation of HPV infection (Kabuki et al., 1997; Gillet et al., 2012; Stoyancheva et al., 2014). Therefore, vaginal *Lactobacillus* spp. play a significant impact in the persistence or regression of the virus and subsequent disease (Petrova et al., 2013; Brotman et al., 2014; DiGiulio et al., 2015; Mitra et al., 2016). Invasive cervical cancer patients exhibit decreased *Lactobacillus* spp., increased *Fusobacterium* spp., and increased overall bacterial diversity and richness (Lin et al., 2020). *Fusobacterium* predominance is more prevalent in individuals with invasive cervical cancer, where it is found to be related with elevated levels of IL-4 and transforming growth factor (TGF)-β1 mRNA, indicating its immunosuppressive effect in the microenvironment of the invasive cervical cancer (Audirac-Chalifour et al., 2016). Microbiota dysbacteriosis might increase the apoptosis of cancer cells or might activate immunosuppressive cells, such as dendritic and Treg cells, and cytokines (Nami et al., 2014; Nami et al., 2014; Eslami et al., 2016; Wang et al., 2018). Therefore, dysbacteriosis has lately been associated with cancer progression and treatment responses (Chang and Parsonnet, 2010).

The function of the vaginal flora in HPV-driven disease has been intensively explored. A previous study has found changes in the female genital tract microbial flora to be related to HPV infection and cervical cancer (Nieves-Ramírez et al., 2021). *Sneathia* and *Prevotella* enrichment is highly related to HPV infection and contributes to HPV persistent infection (Di Paola et al., 2017; Łaniewski et al., 2018; Brusselaers et al., 2019). Both BV and cervical intraepithelial neoplasia (CIN) show a similar characteristics of vaginal flora, which present a decrease in *Lactobacilli* abundance, increased diversity and an increase in the predominance of abnormal anaerobic bacteria (Gillet et al., 2012). Disruption of protective microbiota colonization can lead to a weakening of defense mechanisms. Although the field of microbiome about HPV-driven cancers is emerging rapidly, with most studies focusing on characterizing bacterial profiles, a possible association between vaginal flora composition and HPV clearance or progression to cervical dysplasia and cancer has yet to be shown (Mitra et al., 2016; Shannon et al., 2017; Godoy-Vitorino et al., 2018; Lin et al., 2020; Norenhag et al., 2020; Mitra et al., 2020). Given the part of low *Lactobacillus* cases, more detailed community state types (CSTs) of bacteria in addition to *Lactobacilli* might be helpful for vaginal microbiome studies (Cheng et al., 2020).

It has been reported that a vaginal flora dominated by non-*Lactobacillus* species is connected with the risk of HPV infection and persistence (Lee et al., 2013; Mitra et al., 2016; Shannon et al., 2017; Norenhag et al., 2020). Some *Lactobacillus* species like *L. gasseri*, might be helpful for the clearance of HPV (Brotman et al., 2014; Brusselaers et al., 2019). The probiotic can alter the tumor microenvironment. When *Lactobacillus casei*-containing probiotics were administered to HPV-positive women, enhanced HPV clearance were observed (Verhoeven et al., 2013). Oral probiotics might be helpful to preserve normal vaginal flora during antibiotic therapy (Macklaim et al., 2015), but its efficacy varies widely, and may be influenced by many factors, including interruption from the gut local microenvironment and colonization of bacteria. At the very beginning, limited data are available (Li et al., 2020). Probiotics directly applied to the vaginal environment may play a more direct role in vaginal flora, but little information has been obtained in this field. A deeper understanding of vaginal flora will eventually aid in the development of practical and low-cost treatments to reduce the HPV infection (Li et al., 2020). Given the lack of research in this area, more studies are required to elucidate the effect of probiotic therapy on specific microbiota in patients with HPV infection. In this study, vaginal probiotics (mainly *lactobacilli*) were used to treat the patients with HPV infection, and the entire composition of vaginal microbiota was studied. We also discuss the impact of vaginal flora on HPV clearance.

## Methods

### Patients

A participant was eligible if she (a) was 18-60 years old without HPV vaccination, (b) had not undergone a gynecological reproductive surgery such as cervical conization, hysterectomy, appendectomy, hysteroscopy, etc, (c) had no vaginal flushing and had abstained from sex for at least 72 h, (d) had no history of vaginal medication within 3 days, and no systematic use of antibiotics or antifungal drugs, probiotics, antibiotics or glucocorticoids within 1 month, and (e) was HPV-positive upon initial screening. The 21 HPV GenoArray Diagnostic Kit (Chaozhou Hybribio Biochemistry Ltd, China) was used to conducted HPV typing. Genotypes of 21 HPV genotypes (6, 11, 16, 18, 31, 33, 35, 39, 42, 43, 44, 45, 51, 52, 53, 56, 58, 59, 66, 68 and CP8304 (81)) were detected. Healthy women were enrolled from the physical examination center of Chenghai district people’s hospital; all were HPV-negative. Exclusion criteria: (a) had a vaginal lavage or had sexual activity within 72 hours, (b) used probiotic bacteria, antibiotics, or corticosteroids within the past 30 days, (c) with cancer, diabetes, autoimmune diseases and other serious diseases that may affect the results of this study, (d) was pregnant.

A total of 135 participants were included on the basis of the inclusion and exclusion criteria. All procedures for this study were approved by the Research Ethics Committee of the First Affiliated Hospital of Shantou University Medical College (No. 201561).

### Sample collection, DNA extraction, and 16S sequencing

Cervical specimens were collected from female patients between January 2016 and June 2018.

Genomic DNA was extracted from the samples by using the CTAB (Cetyltrimethylammonium Bromide) method. After the detection of purity and concentration of genomic DNA, using genomic DNA diluted with sterile water to 1 ng/μL, specific primers with barcode, New England Biolabs Phusion^®^ High Fidelity PCR Master Mix with GC Buffer (New England Biolabs, USA) and Phusion^®^ High-Fidelity DNA polymerase (New England Biolabs, USA) were used for PCR. After mixing in equal amounts according to the PCR product concentration, electrophoresis purification was performed on a 1× TAE 2% agarose gel, shearing to obtain the band of interest, and the sheared target bands were recovered using a DNA purification kit (DP214, Tiangen, China). The construction of library was performed using Ion Plus Fragment Library Kit 48 (Thermo Fisher, USA), which was sequenced using an Ion S5™XL (Thermo Fisher, USA) after Qubit quantification and library detection.

### Bioinformatics analyses

Low-quality data was removed by using Cutadapt (V1.9.1) (Langille et al., 2013). Then barcode and primer sequences were trimmed. Clean reads were obtained after detecting and removing chimeric sequences (Rognes et al., 2016) from raw data by using VSEARCH (Martin, 2011). All clean reads of all samples were clustered as Operational Taxonomic Units (OTUs) using the UPARSE algorithm (UPARSE v7.0.1001) (Haas et al., 2011) by default with 97% identity. The annotation of OTUs representative sequences were performed by using Mothur method and SSUrRNA database (Wang et al., 2007) of SILVA132 (Edgar, 2013) (threshold was set at 0.8~1) obtain species information and species abundance at each taxonomic level. MUSCLE (Quast et al., 2013) (Version 3.8.31), software was used to do fast sequence alignment and then the process of homogenization was conducted. ALPHA diversity indices and the UniFrac distance were calculated by using QIIME software (Version 1.9.1) (Caporaso et al., 2010).

### Statistical analyses

Alpha diversities were visualized in the box plot using the package “ggplot2” in R software (Version 4.3.0). Principal co-ordinates analysis (PCoA) was conducted on basis of the unweighted UniFrac distance matrix using “vegan”, “Parseq”, and “ggplot2” packages. PERMANOVA analysis was conducted using the “vegan” package. Linear discriminant analysis Effect Size (LEfSe) analysis was done by using LEfSe software, with the default value of the linear discriminant analysis (LDA) score being 2. The Chord diagram was performed using the “circlize” package in R. Kruskal-Wallis tests were employed to analyze differences in microbial α diversity among multiple groups. If *p* < 0.05, Dunn’s Test was used to perform pairwise comparisons between each independent group. Measurement data for demographic and clinical characteristics were analyzed using the Kruskal-Wallis test, while the ordinal categorical variable was analyzed using the Wilcoxon rank-sum test. The association between HPV and age, cleaning degree of the vagina, menopause, contraception, non-menstrual bleeding, the number of pregnancies, births, miscarriages, and the number of white blood cells were analyzed by Spearman rank correlation analysis in SPSS 26.0.

## Results

### Patient’s characteristics

**Figure 1:**
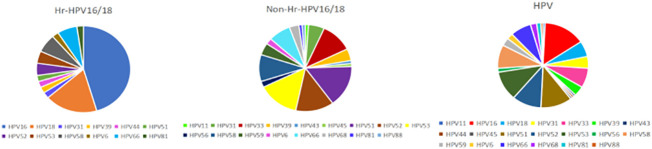
Prevalence of different HPV subtypes in infected groups. Hr-HPV16/18 (n = 28): HPV16/18 high-risk infection group; non-Hr-HPV16/18 (n = 62): non-HPV16/18 high-risk infection group; HPV: HPV-infected group (n = 90).

In total, 135 participants were included in the research (**Figure S1**), including 45 normal controls and 90 patients with HPV infection, in which the HPV subtypes with the highest frequencies were HPV16 (22.22%, n = 20), HPV51 (16.67%, n = 15), HPV53 (16.67%, n = 15), and HPV52 (15.56%, n = 14). The samples were divided into two groups based on whether the infected HPV type was HPV16/18 (Hr-HPV16/18, n = 28) or was non-HPV16/18 (non-Hr-HPV16/18, n = 62). The morbidity of HPV16 was 71.43% (n = 20), and for HPV18 was 28.57% (n = 8) in the Hr-HPV16/18 group, while in the non-Hr-HPV16/18 group, the main types were HPV51, HPV52, and HPV53, and their morbidities were 22.58% (n = 14), 19.35% (n = 12), and 20.97% (n = 13), respectively (**Figure 1**). The difference in age among the three groups (controls, 36 (IQR = 17); Hr-HPV16/18, 37 (IQR = 18); non-Hr-HPV16/18, 41.5 (IQR = 10.75), *p* > 0.05) was not statistically significant. Only a small number of women in the three groups were in menopause; the ratio of women in menopause was 13.33% (n = 6) in the controls, 7.14% (n = 2) in Hr-HPV16/18, and 17.74% (n = 11) in non-Hr-HPV16/18 patients, respectively. But patients with non-menstrual bleeding had significant differences among the Hr-HPV16/18, non-Hr-HPV16/18, and the controls (*p* < 0.01). No positive cases of BV were found, but some cases (n = 14) have tested positive for Ureaplasma urealyticum, M. hominis, and Chlamydia trachomatis, and only one positive case of trichomoniasis. Other parameters, including squamous intraepithelial disease (SIL) grade, menopause, and IUD/tubal ligation/condom, the number of pregnancies, births, miscarriages, and white blood cells, were measured and showed no significant difference (*p* > 0.05). Detailed demographics and clinical characteristics of participants were showed in **Table 1**.

**Table 1:** Descriptive characteristics of all samples included in the study.

Variable	Subcategory	Controls (n = 45)	Hr-HPV16/18 (n = 28)	Non-Hr-HPV16/18 (n = 62)	*p*
Age, yrs		36 (IQR = 17)	37 (IQR = 18)	41.5 (IQR = 10.75)	0.134
SIL grade	Low grade	2 (4.44%)	1 (3.57%)	9 (14.52%)	0.119
	High grade	1 (2.22%)	4 (14.29%)	6 (9.68%)	
	ASC-US	2 (4.44%)	10 (35.71%)	23 (37.10%)	
	ASC-H	1 (2.22%)	4 (14.29%)	0 (0.00%)	
Menopause	YES	6 (13.33%)	2 (7.14%)	11 (17.74%)	0.405
	NO	39 (86.67%)	26 (92.86%)	51 (82.26%)	
Non-menstrual bleeding	YES	30 (66.67%)	9 (32.14%)	20 (32.26%)	<0.001
	NO	15 (33.33%)	19 (67.86%)	42 (67.74%)	
IUD/Tubal ligation/condom	YES	20 (44.44%)	10 (35.71%)	24 (38.71%)	0.318
	NO	25 (55.56%)	18 (64.29%)	38 (61.29%)	
Trichomonad	YES	1 (2.22%)	0 (0.00%)	0 (0.00%)	
	NO	44 (97.78%)	28 (100.00%)	62 (100.00%)	
No. of pregnancies		4 (IQR = 2)	3 (IQR = 2)	3 (IQR = 2)	0.596
No. of births		2 (IQR = 1.5)	2 (IQR = 1)	2 (IQR = 1)	0.913
No. of miscarriages		1 (IQR = 2)	0 (IQR = 1)	0 (IQR = 2)	0.786
No. of white blood cells		6.11 (IQR = 4.36)	7.34 (IQR = 1.75)	5.60 (IQR = 2.62)	0.076
Cleaning degree of vagina	I	4 (8.89%)	4 (14.29%)	9 (14.52%)	0.101
	II	25 (55.56%)	22 (78.57%)	40 (64.52%)	
	III	6 (13.33%)	1 (3.57%)	3 (4.84%)	
	IV	3 (6.67%)	0 (0.00%)	2 (3.23%)	
UU (Ureaplasma urealyticum)	+	1 (2.22%)	3 (10.71%)	6 (9.68%)	
	–	0 (0.00%)	1 (3.57%)	3 (4.84%)	
MH (M. hominis)	+	1 (2.22%)	2 (7.14%)	4 (6.45%)	
	–	0 (0.00%)	2 (7.14%)	5 (8.06%)	
CT (Chlamydia trachomatis)	+	0 (0.00%)	1 (3.57%)	2 (3.23%)	
	–	1 (4.44%)	3 (10.71%)	7 (11.29%)	
Cervical intraepithelial neoplasm		3 (6.67%)	2 (7.14%)	6 (9.68%)	

Hr-HPV16/18, HPV16/18 high-risk infection group; Non-Hr-HPV16/18, non-HPV16/18 high-risk infection group; SIL, squamous intraepithelial lesion; ASC-US, atypical squamous cells of undetermined significance; ASC-H, atypical squamous cells: cannot exclude high-grade squamous intraepithelial lesion; IUD, intrauterine device.

### Vaginal bacterial diversity in patients with HPV and non-HPV infection

**Figure 2:**
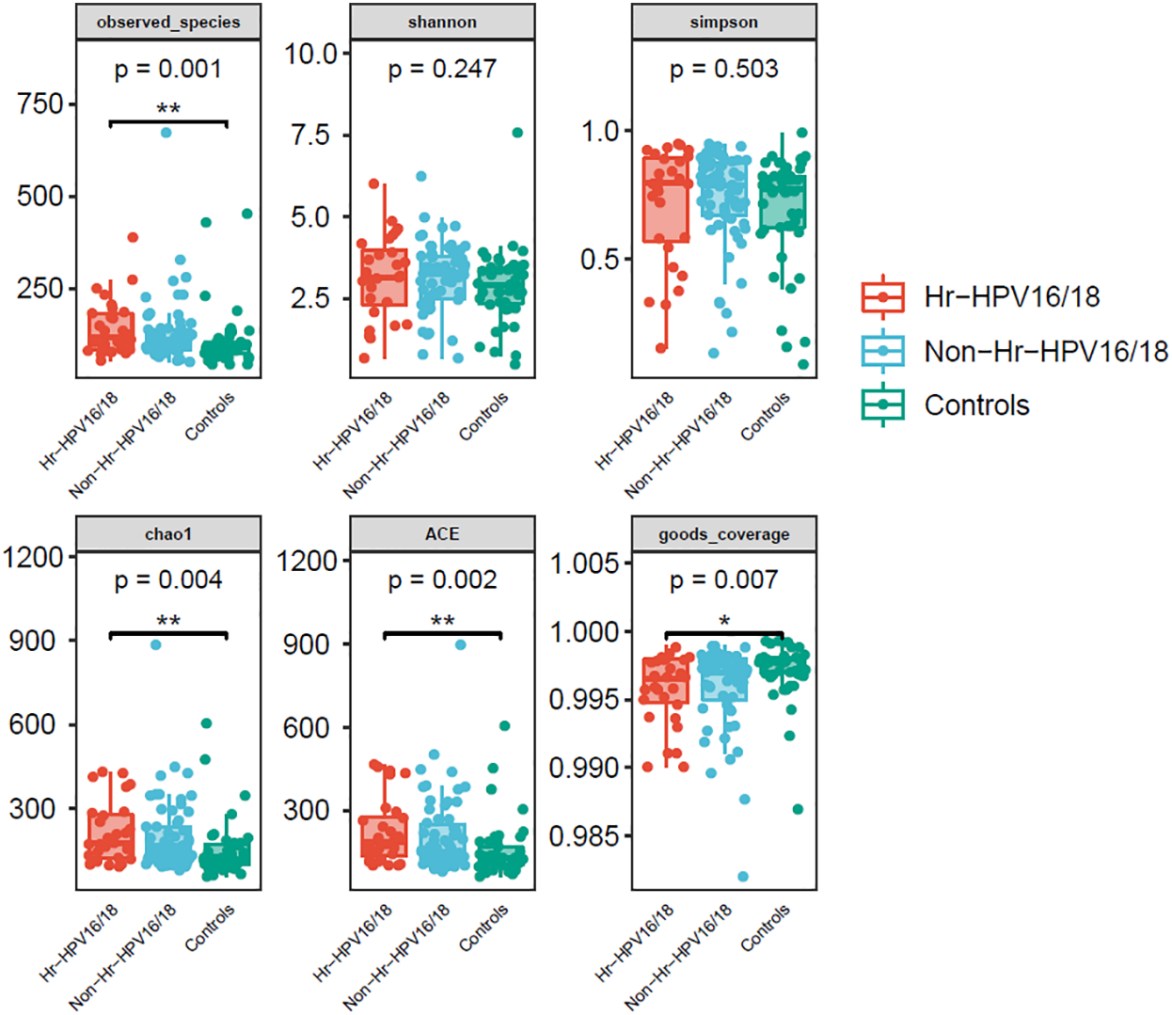
Comparison of vaginal microbial alpha diversity index (observed - species, Shannon, Simpson, Chao1, ACE, good - coverage) in infected and healthy individuals. The *p*-value on the top indicates the overall difference among three groups calculated using the Kruskal-Wallis nonparametric test method, and the asterisks on the top indicate a statistically significant difference between the two groups calculated using Dunn’s test (*p < 0.05, * *p < 0.01).

**Figure 3:**
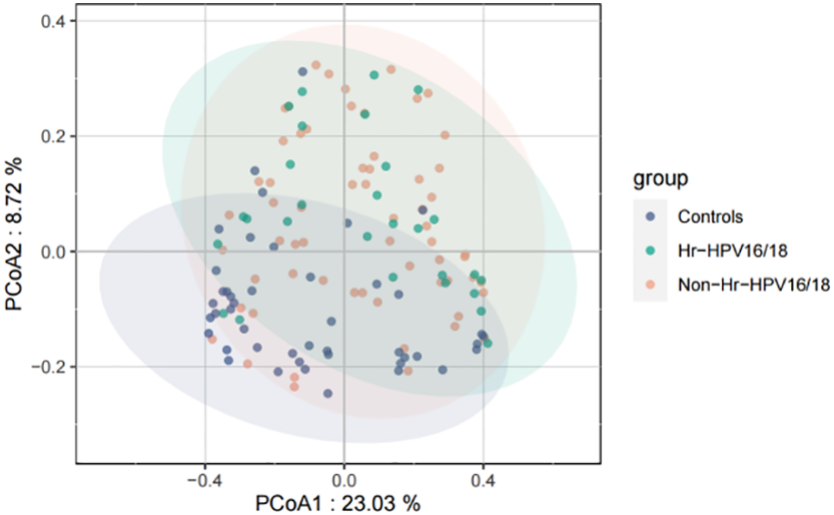
Principal coordinates analysis (PCoA) of variation in beta diversity of human vaginal bacterial communities in infected and healthy individuals based on unweighted UniFrac phylogenetic distance. Hr-HPV16/18 (n = 28): HPV16/18 high-risk infection group; Non-Hr-HPV16/18 (n = 62): non-HPV16/18 high-risk infection group; controls (n = 45): non-HPV infection group.

High-quality classifiable 16S ribosomal DNA sequences were acquired, with 77,391 clean reads per sample. Bacterial communities and their alpha diversity were measured. Our results showed that there was a significantly higher observed species, and ACE and Chao1 scores in the Hr-HPV16/18 groups than those in controls (**Figure 2**), while there was no significant difference in Shannon and Simpson indices between the two groups (*p* > 0.05), and there was no significant difference between the non-Hr-HPV16/18 groups and the controls (*p* > 0.05). These results show a higher microbial diversity in the Hr-HPV16/18 groups. No clear separation of samples between the HPV-uninfected and infected groups was showed in PCoA analysis, indicating there were no significant similarity differences in microbial composition among the three groups (**Figure 3**).

### Comparison of bacteria at the phylum, genus, and species levels between the HPV-infected group and controls

**Figure 4:**
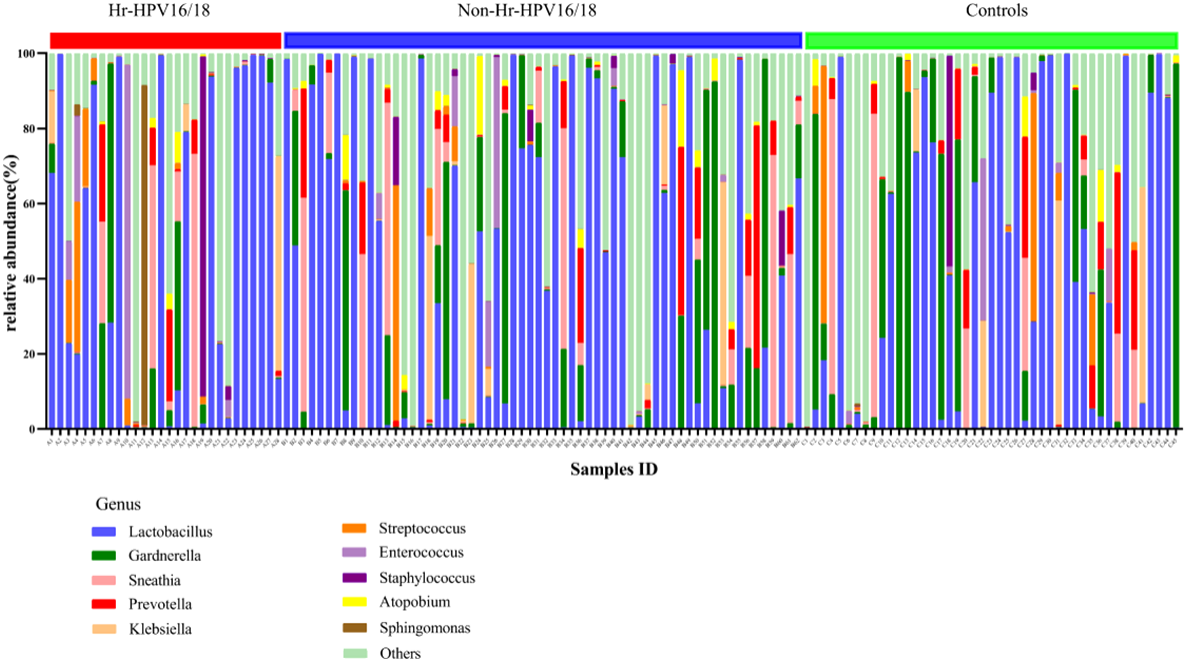
Vaginal flora at the genus level in infected and healthy individuals.

**Figure 5:**
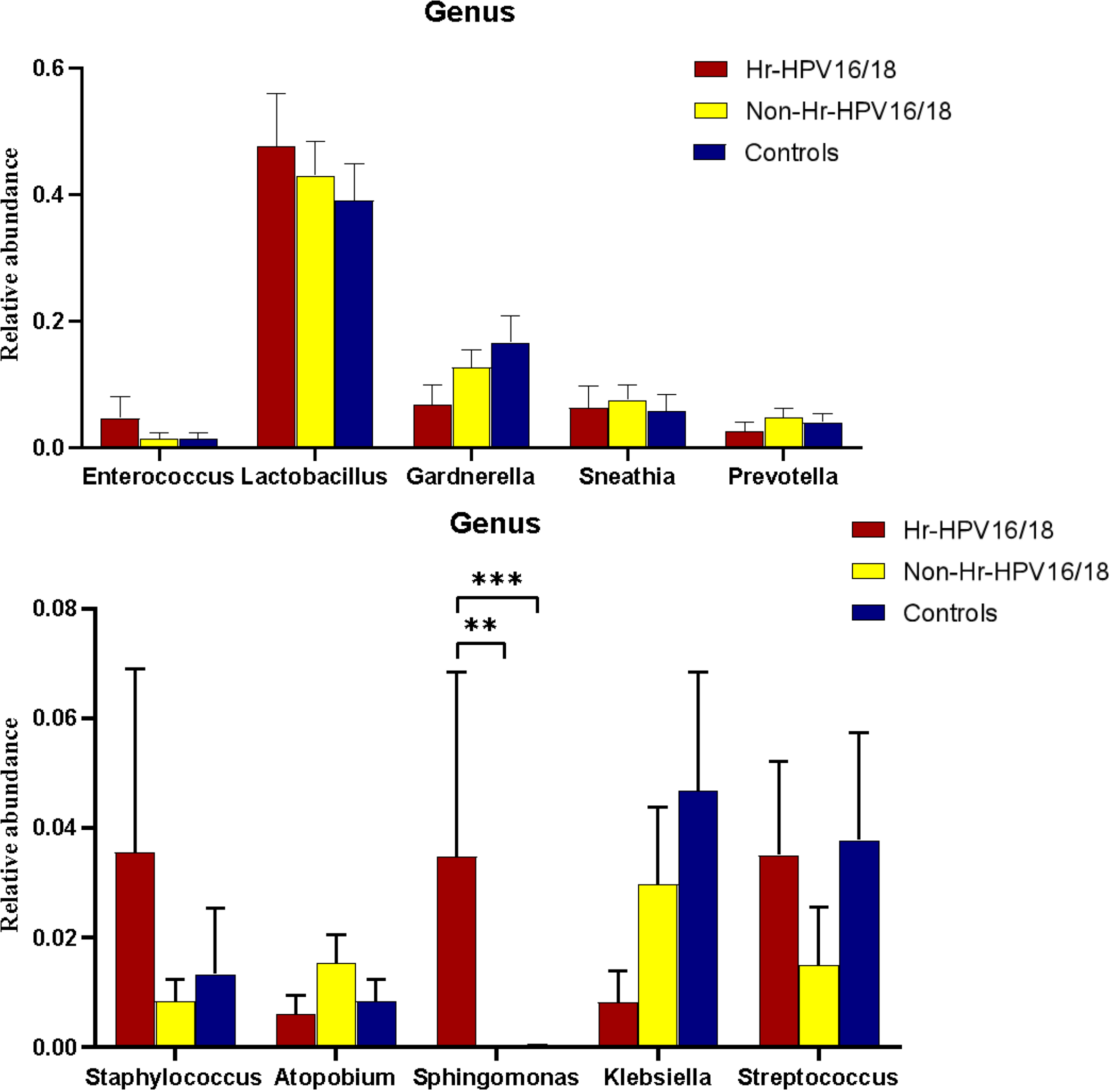
Comparison of relative abundance of the top 10 vaginal microflora genera between the Hr-HPV16/18 (n = 28), non-Hr-HPV16/18 (n = 62), and healthy (n = 45) individuals. *p* - values of 0.05 indicate a statistically significant difference, **p < 0.01, ***p < 0.001.

In vaginal flora, Firmicutes was the most predominant phylum in the healthy individuals and HPV-infected patients, followed by Proteobacteria, Actinobacteria, Fusobacteria, and Bacteroidetes. There was no significant difference between the HPV-infected group and control. Then, we characterized the differences in vaginal flora among the three groups at the genus level. The top 10 bacteria in the three groups (Hr-HPV16/18 vs. non-Hr-HPV16/18 vs. controls) were: *Lactobacillus* spp. (46.47% vs. 43.46% vs. 39.04%, *p* > 0.05); *Gardnerella* spp. (6.60% vs. 12.69% vs. 17.07%, *p* > 0.05); *Sneathia* spp. (6.15% vs. 7.59% vs. 5.94%, *p* > 0.05); *Prevotella* spp. (2.61% vs. 4.73% vs. 4.17%, *p* > 0.05); *Klebsiella* spp. (2.83% vs. 2.93% vs. 3.62%, *p* > 0.05); *Streptococcus* spp. (3.38% vs. 1.48% vs. 3.94%, *p* > 0.05); *Enterococcus* spp. (4.56% vs.1.49% vs. 1.48%, *p* > 0.05); *Staphylococcus* spp. (3.43% vs. 0.83% vs. 1.39%, *p* > 0.05); *Atopobium* spp. (0.59% vs. 1.51%, vs. 0.89%, *p* < 0.05), and *Sphingomonas* spp. (3.36%, vs. 0.01% vs.0.02%, *p* < 0.05) (**Figures 4**, **5**).

**Figure 6:**
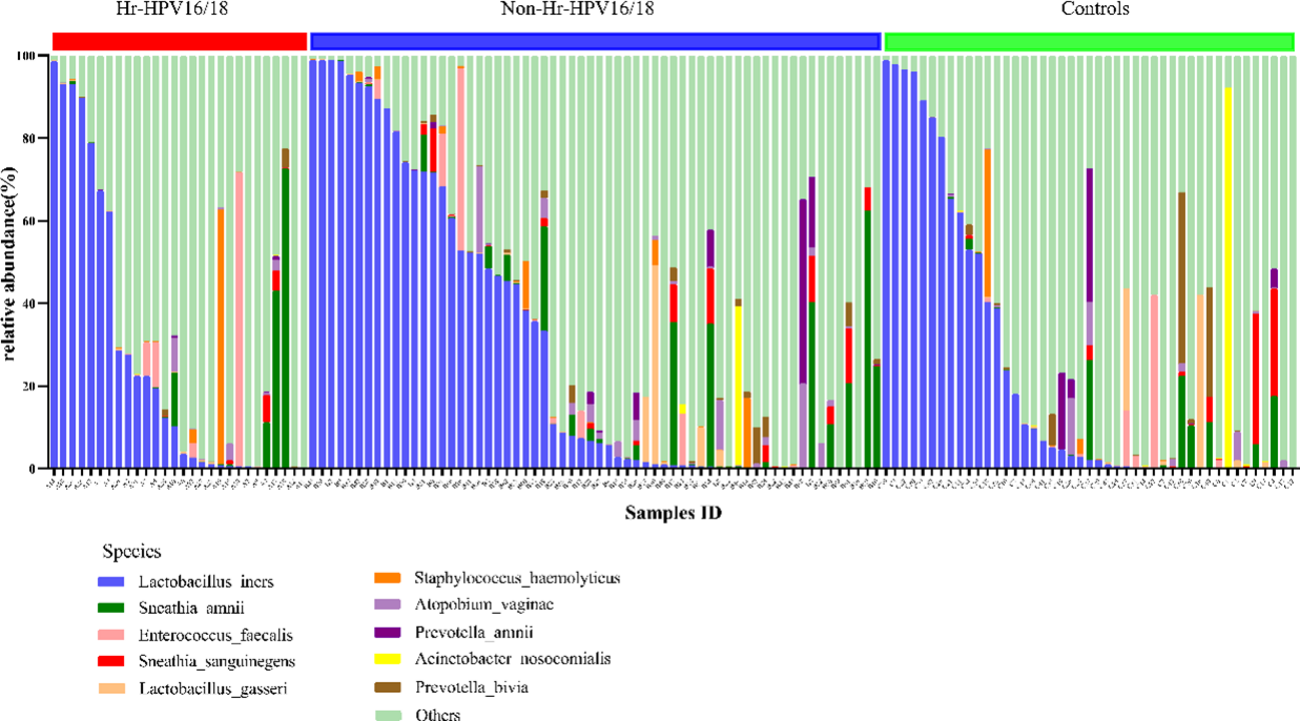
Vaginal flora at the species level of Hr-HPV16/18 (n = 28), non-Hr-HPV16/18 (n = 62), and controls (n = 45).

At the bacterial species level, the top 10 species in three groups (Hr-HPV16/18 vs. non-Hr-HPV16/18 vs. controls) were: *L. iners* (26.29% vs. 30.16% vs. 23.27%, *p* > 0.05); *Sneathia amnii* (*S. amnii*) (5.08% vs. 4.69% vs. 2.11%, *p* < 0.05); *Enterococcus faecalis* (*E. faecalis*) (3.37% vs. 1.34%, vs. 1.38%, *p* > 0.05); *Sneathia sanguinegens* (*S. sanguinegens*) (0.46% vs. 1.29% vs. 1.56%, *p* > 0.05); *L. gasseri* (0.09% vs. 1.33% vs. 1.72%, *p* < 0.05); *Staphylococcus haemolyticus* (*S. haemolyticus*) (2.38% vs. 0.71% vs. 0.91%, *p* > 0.05); *Atopobium vaginae* (*A. vaginae*) (0.59% vs. 1.51% vs. 0.88%, *p* < 0.05); *Prevotella amnii* (*P. amnii*) (0.06% vs. 1.34% vs.1.34%, p>0.05); *Acinetobacter nosocomialis* (*A. nosocomialis*) (0.02%; vs. 0.67% vs. 2.08%, *p* > 0.05), and *Prevotella bivia* (*P. bivia*) (0.23% vs.0.64% vs.1.79%, *p* < 0.05) (**Figure 6**).

### Features of vaginal microflora in individuals infected with single, dual, or multiple HPVs

**Figure 7:**
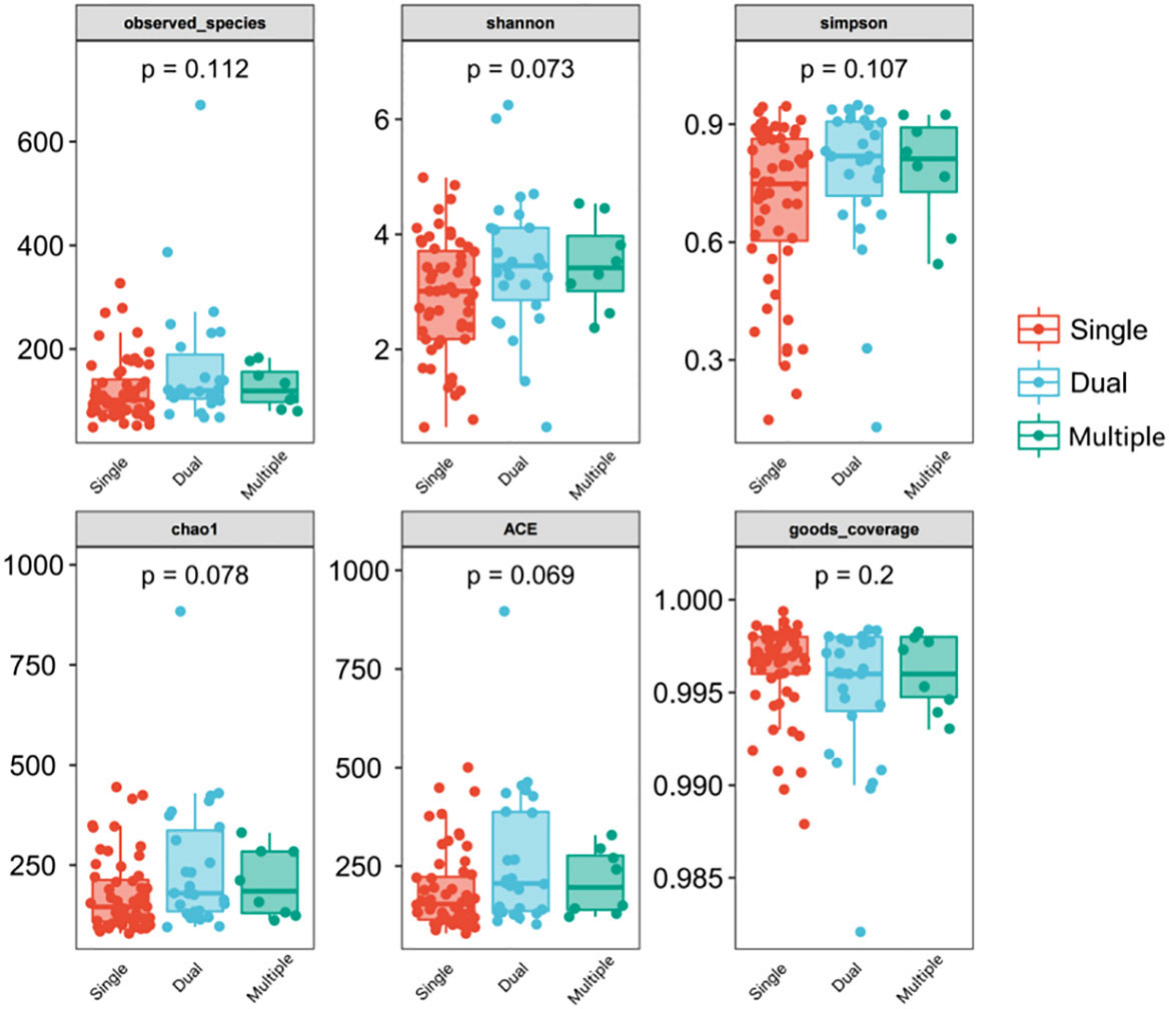
Comparison between alpha diversity index (observed - species, Shannon, Simpson, Chao1, ACE, good-coverage) of infected and healthy individuals. The *p*-value on the top indicates the overall difference among three groups calculated using the Kruskal-Wallis nonparametric test method, and the asterisks on the top indicate a statistically significant difference between the two groups calculated using Dunn’s test (* *p* < 0.05, ** *p* < 0.01). Single (n = 56): infected with a single HPV subtype; dual (n = 26): infected with two HPV subtypes; Multiple (n = 8): infected with three or more HPV subtypes.

**Figure 8:**
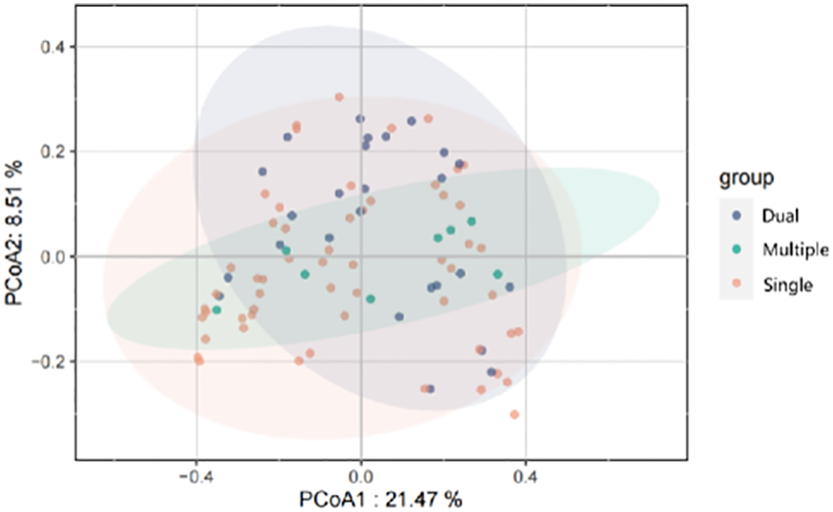
Principal-coordinates analysis (PCoA) of variation in beta diversity of human vaginal bacterial communities in infected and healthy individuals, based on unweighted UniFrac phylogenetic distance.

**Figure 9:**
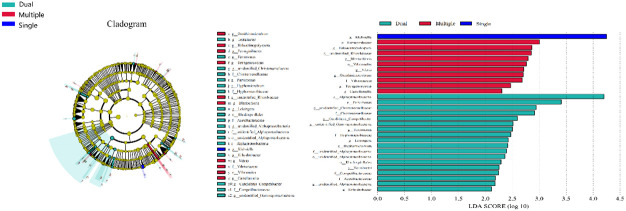
LEfSe analysis comparing microbial variations at the genus level in patients infected with one, two, or multiple HPV subtypes. LEfSe cladogram representing differentially abundant taxa (*p* < 0.05). LDA scores as calculated by LEfSe of taxa are differentially abundant among groups. Only taxa with LDA scores of >2 were presented.

We divided patients into three groups: infected by a single HPV type (n = 56), infected by two HPV types (n = 26), and infected by multiple HPV types (n = 8). We further analyzed whether vaginal microbial diversity would be influenced by the number of infected HPV types. Women infected with multiple HPV types tended to have higher alpha diversity than those infected with a single HPV type, but the difference was not statistically significant (*p* > 0.05) (**Figures 7**, **8**). To identify bacteria specifically linked with HPV infection status, LDA with effect size (LEfSe) modeling was conducted (**Figure 9**). The larger LDA indicated the greater difference of the species. In the single HPV type group, the predominant species was *Klebsiella* (*p* < 0.05). In the dual HPV type group, the predominant species were *Parvimonas*, *unidentified Christensenellacea*, *Candidatus competibacter*, *unidentified Gammaproteobacteria*, *Terrimonas*, *Leisingera*, *Hyphomicrobium*, *Terrabacter*, *unidentified Alphaproteobacteria* and *Enhydrobacter* (*p* < 0.05). In the multiple HPV type group, the predominant species were *Ferruginibacter*, *Haloactinopolyspora*, *unidentified Rhizobiaceae*, *Blastochloris*, *Vibrio*, *Ornithinimicrobium*, *Tetragenococcus*, and *Castellaniella*.

### Vaginal flora features in patients with HPV clearance

**Figure 10:**
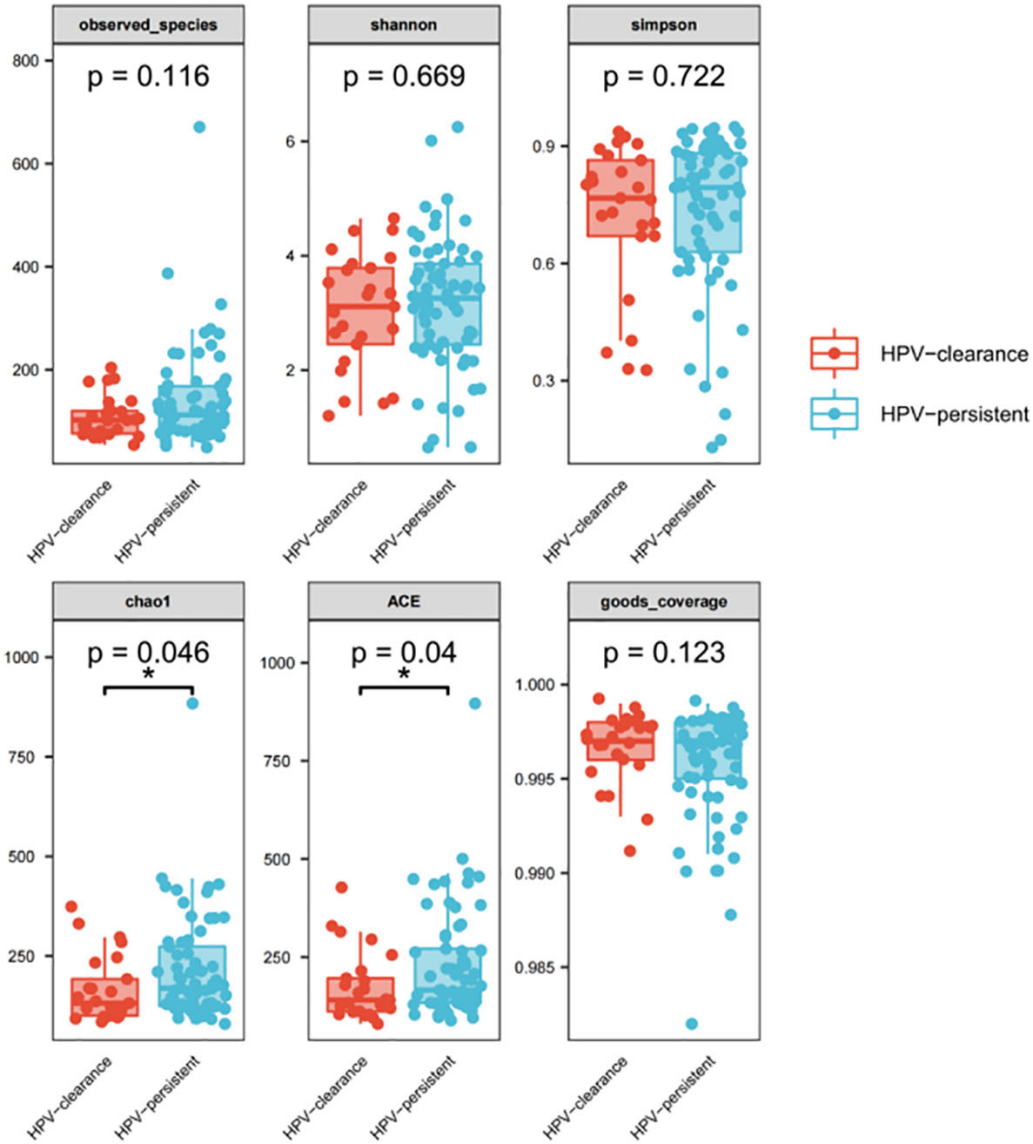
Alpha diversity index (observed - species, Shannon, Simpson, Chao1, ACE, good-coverage) in the HPV-cleared and HPV-persistent groups. The *p*-value on the top indicates the overall difference among three groups calculated using the Kruskal-Wallis nonparametric test method, and the asterisks on the top indicate a statistically significant difference between the two groups calculated using Dunn’s test (* *p* < 0.05, ** p < 0.01).

We further analyzed whether vaginal flora affects HPV clearance. There were 26 patients who had HPV clearance within a year, with most turning negative within six months. Analysis of vaginal flora characteristics showed the women with HPV clearance had significantly lower bacterial diversity, with scores of 0.046 for Chao1, and 0.04 for ACE diversity, respectively (**Figure 10**).

**Figure 11:**
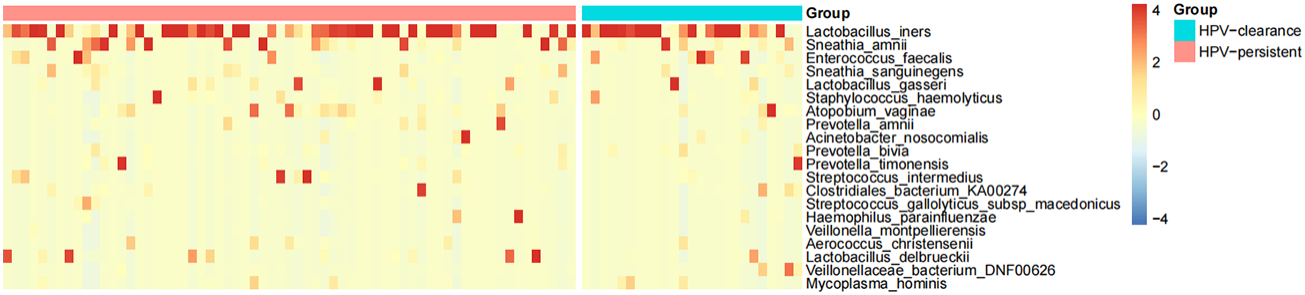
Heat map analysis of bacterial species found in the vaginal flora of 90 women. Each vertical line represents one sample. Different colors indicate relative abundance: red represents a high proportion and blue represents a low proportion. HPV-clearance: patients who cleared HPV; HPV-persistent: patients who did not clear HPV.

**Figure 12:**
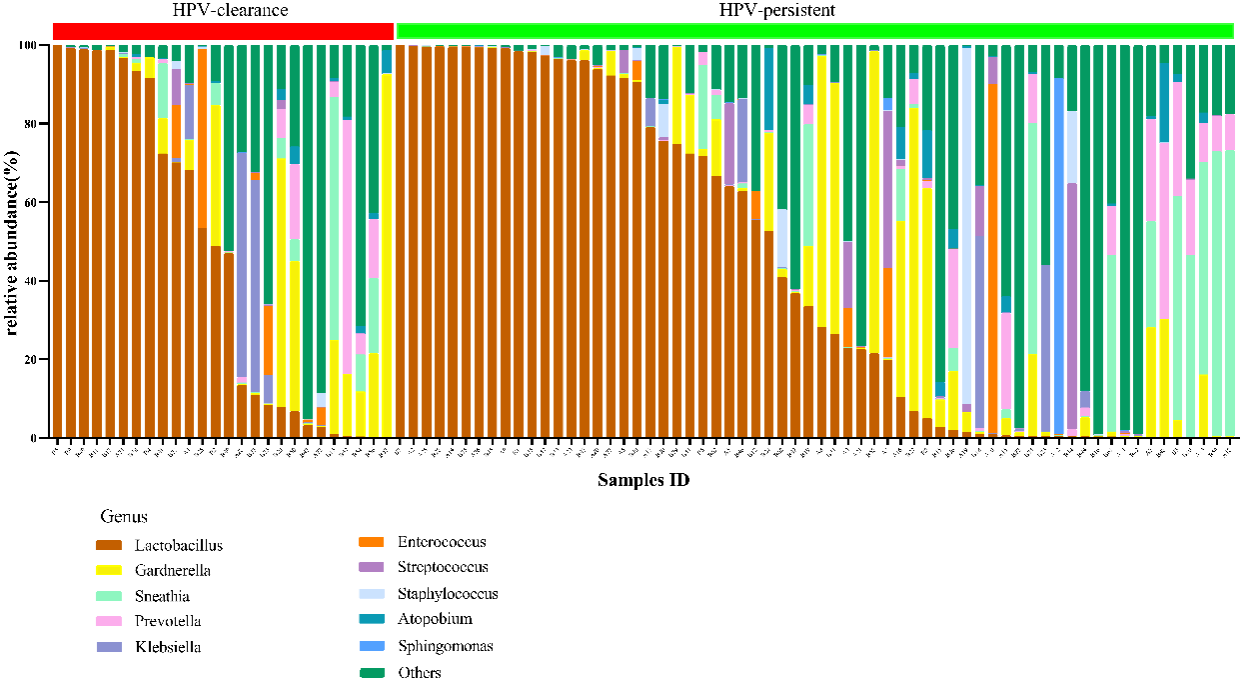
Vaginal flora at the genus level of HPV-cleared (n = 26) and HPV-persistent (n = 64) patients.

The top 20 bacteria were analyzed by heat mapping (**Figure 11**). At the phylum level, no significant changes were indicated between patients with HPV clearance and HPV persistence, for Firmicutes (53.53% vs. 57.55%, *p* > 0.05), Proteobacteria (21.90% vs. 15.24%, *p* > 0.05), Actinobacteria (13.59% vs. 12.09%, *p* > 0.05), Fusobacteria (4.76% vs. 8.33%, *p* > 0.05), and Bacteroidetes (5.40% vs. 5.51%, *p* > 0.05). At the genus level, no significant changes were observed for *Lactobacillus* (45.79% vs. 43.83%, *p* > 0.05), *Gardnerella* (12.67% vs. 10.03%, *p* > 0.05), *Sneathia* (4.75% vs. 8.11%, *p* > 0.05), *Prevotella* (4.55% vs. 3.87%, *p* > 0.05), *Klebsiella* (5.16% vs. 1.99%, *p* > 0.05), *Enterococcus* (3.22% vs. 2.12%, *p* > 0.05), *Streptococcus* (0.50% vs. 2.71%, *p* > 0.05), *Staphylococcus* (0.28%, vs. 2.19%, *p* > 0.05), *Atopobium* (0.77% vs. 1.40%, *p* > 0.05), and *Sphingomonas* (0.01% vs. 1.48%, *p* > 0.05) (**Figure 12**). At the species level, between patients who had cleared HPV vs. patients with persistent HPV, no changes were observed for *L. iners* (32.44% vs. 27.54%, *p* > 0.05), *S. amnii* (2.73% vs. 5.65%, *p* > 0.05), *E. faecalis* (2.87% vs. 1.61%, *p* > 0.05), *S haemolyticus* (0.26% vs. 1.62%, *p* > 0.05), *A. vaginae* (0.77% vs.1.40%, *p* > 0.05), *Streptococcus intermedius* (*S. intermedius*) (0.31% vs. 1.33%, *p* > 0.05), *S. sanguinegens* (0.84% vs. 1.11%, *p* > 0.05), *Prevotella timonensis* (*P. timonensis*) (1.61% vs. 0.70%, *p* > 0.05), *P. amnii* (0.13% vs. 1.27%, *p* > 0.05), and *L. gasseri* (0.67% vs. 1.05%, *p* > 0.05).

**Figure 13:**
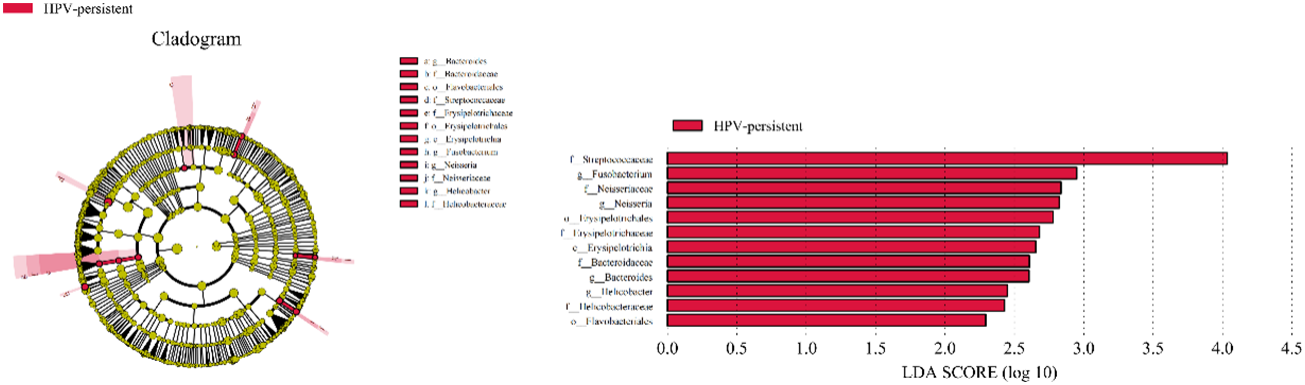
LEfSe analysis comparing microbial variations at the genus level in infected and healthy individuals. LEfSe cladogram representing differentially abundant taxa (*p* < 0.05). LDA scores as calculated by LEfSe of taxa are differentially abundant among groups. Only taxa with LDA scores of >2 are presented.

To identify bacteria specifically linked with HPV clearance, LEfSe modeling was conducted (**Figure 13**). Our results showed that patients who had developed a persistent HPV infection had significantly higher levels of Erysipelotrichia (class level), Bacteroidaceae, Erysipelotrichaceae, Helicobacteraceae, Neisseriaceae, Streptococcaceae (family level), Erysipelotrichales, Flavobacteriales (order level), and *Fusobacterium*, *Bacteroides*, *Neisseria*, and *Helicobacter* (genus level) than patients who had cleared HPV (*p* < 0.05).

### Factors associated with HPV infection

**Figure 14:**
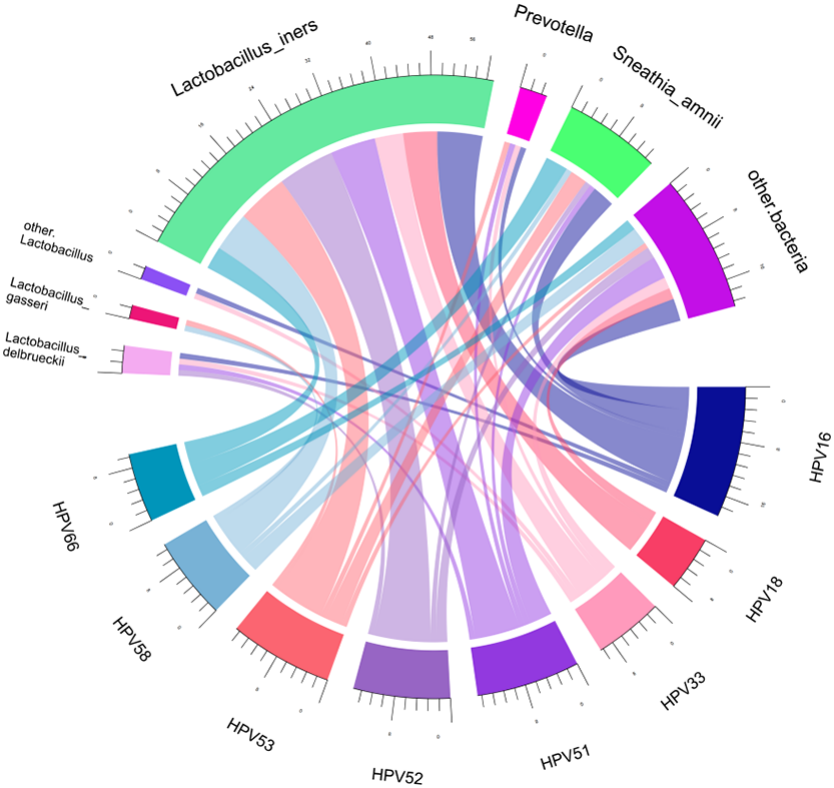
Chord diagram shows the relationship between HPV subtypes and dominant vaginal bacteria. The width of the strings (connecting lines) in the chord diagram shows the extent and proportion of the association between different HPV subtypes and dominant vaginal bacteria. The wider the width of the connecting lines, the higher the proportion. Different colors distinguish between different relationships.

Spearman correlation analysis was exploited to analyze the correlation between HPV infection and clinical biomarkers. Our results showed that vaginal cleanliness and non-menstrual bleeding were two related factors, with correlation coefficients of ρ = 0.195 (*p* < 0.05) and ρ = 0.327 (*p* < 0.05), respectively. The main HPV subtypes and the dominant bacteria detected in all patient samples were selected for a chord diagram (**Figure 14**). Each HPV subtype was linked to a dominant bacterium. The wider the link was, the larger the number of dominant bacteria in patients of this subtype. *L. iners* was the bacterial species most connected with HPV subtypes. The second was the *S. amnii*, and the third was the *Prevotella*. In addition, we found that the dominant bacteria with the highest prevalence in HPV-positive samples were *L. iners* – dominant (n = 49, 54.44%) and *S. amnii*-dominant (n = 10, 11.11%). The top 3 dominant bacteria in the HPV-persistent group were *L. iners* (n = 34, 53.13%), *S. amnii* (n = 9, 14.06%), and *L. delbrueckii* (n = 3, 4.69%).

## Discussion

The vaginal flora is an important factor in modulating the vaginal mucosa microenvironment against viral infections (Nieves-Ramírez et al., 2021). Viral infection may also disturb the normal structure and composition of the vaginal flora. In this study, we found that bacterial components vary between the HPV infectious subgroups and healthy controls. The dominant bacteria with the highest prevalence in HPV-positive group were *L. iners*, *S. amnii*, and *Prevotella*. Particularly, *S. amnii* was significantly higher, *L. gasseri, P. bivia*, and *A. vaginae* were significantly lower in the Hr-HPV 16/18 group than those in healthy individuals. This decrease in the predominating protective bacteria leads to the increase of vaginal pH levels, the weakening of pathogenic defense ability and the damage of mucosal barriers (Gillet et al., 2012; Łaniewski et al., 2018). The differences in the composition of vaginal flora may be the basis for dysbiotic patterns associated with HPV infection and cervical cancer in different female populations (Bychkovsky et al., 2016; Curty et al., 2017; Romero-Morelos et al., 2019; Nieves-Ramírez et al., 2021).

The vaginal flora is a complicated ecosystem affected by a variety of factors, including environment, host, and ethnicity (Serrano et al., 2019; Moosa et al., 2020). It has also been reported that different ethnic groups have different characteristics of the cervicovaginal microbiota (Casey et al., 2012). The prevalence of *Lactobacillus* spp. as the dominant microbiota is higher in Caucasian and Asian women compared to Hispanic and Black women (Ravel et al., 2011; Anahtar et al., 2015). These differences may be resulted from genetic factors affecting mucosal immunity or metabolic pathways, leading to preferred conditions for specific species, or they may the consequence of differences in different hygiene practices (Mitra et al., 2016). In this study, all participants are from Chinese, which allows us to limit confounders due to race-ethnic diversity that may affect the vaginal flora (Serrano et al., 2019). Co-variates that may impact on the vaginal flora include smoking status, time within the menstrual cycle, sexual behavior, use of hormonal contraceptives or copper intrauterine devices, as well as ethnic background (Cherpes et al., 2008; Srinivasan et al., 2010; Gajer et al., 2012; Romero et al., 2014). Our results show that vaginal flora is dominated by *Lactobacillus*, followed by *Gardnerella, Sneathia, Prevotella, Klebsiella, Streptococcus, Enterococcus, and Staphylococcus*, either in HPV-infected or in healthy individuals. Several vaginal microbes, such as increased *Gardnerella, Fusobacteria, Bacillus cohnii, Dialister, Prevotella*, and *Mycoplasma*, are associated with dysbiosis that would lead to instability in the microenvironment, which in turn may allow key risk factors to have an impact on cervical cancer (Gao et al., 2013; Ritu et al., 2019; Usyk et al., 2020; Kovachev, 2020).. In our study, the bacterial genera *Sphingomonas* showed a significant difference between the Hr-HPV16/18 group and the healthy controls. In the top 10 bacterial species, the abundance of *S. amnii*, *E. faecalis*, and *S. haemolyticus* were two-fold higher, and *P. amnii*, *A. nosocomialis*, *S. sanguinegens*, *L. gasseri* were two-fold lower in the Hr-HPV 16/18 group than in the controls. *L. gasseri*, rather than *L. iners*, was significantly different among the three groups. *Sneathia* spp. has frequently been associated with HPV positivity (Lee et al., 2013), but its different species varied in HPV-infected subgroups. Therefore, the pattern of flora associated with HPV infection may be unique in different populations. Identifying changes in bacterial composition of HPV-associated cervical cancers may offer new ideas into potential target populations and potential biomarkers of disease and disease-state (Lin et al., 2020).

The local cervical microenvironment can also affect the natural course of HPV infection (Castle and Giuliano, 2003). The previous study revealed that transient and persistent HPV16 infections, comparing with no HPV infection, are related to vaginal flora dominated by non-*Lactobacillus* species (Berggrund et al., 2020). We then studied whether specific vaginal bacteria are associated with HPV clearance. Our results show that bacterial genera (*Sneathia, Streptococcus, Staphylococcus* and *Sphingomonas*), and species (*S. amnii, S. haemolyticus, S. intermedius*, and *P. amnii*), were observed significantly higher in the persistent HPV group than those in the cleared HPV group. LDA analysis showed that *Fusobacterium*, *Bacteroides*, *Neisseria*, and *Helicobacter* are characteristic bacterial genera that are significantly different between patients with persistent HPV and in patients who cleared HPV. Women with a certain specific composition of vaginal flora might be more susceptible to HPV infection or show faster progression of dysplasia (Norenhag et al., 2020). Distinguishing bacterial features associated with HPV clearance in patients will be helpful for early intervention and reversal of persistent infection, which will contribute to reducing the incidence of cervical cancer.

Vaginal *Lactobacilli* can exert vaginal protection through multiple mechanisms. For example, *Lactobacilli* can offer broad-spectrum protection by producing lactic acid, bacteriocins, and biosurfactants, and forming barriers against pathogenic infections in the vaginal microenvironment by adhering to the mucosa (Mitra et al., 2016; Piyathilake et al., 2016; Łaniewski et al., 2019; Ilhan et al., 2019; Borgogna et al., 2020). For treatment options, the addition of exogenous probiotics, i.e., *Lactobacillus* can improve the treatment of cervicovaginal dysbiosis and persistent HPV infections (Qingqing et al., 2021). In comparision with short-term use, long-term use of vaginal probiotics containing *Lactobacillus* spp. is related with increased clearance of HPV (Palma et al., 2018). However, the therapeutic effect varies greatly. Among the main components of a healthy vaginal flora is the presence of *Lactobacillus* spp., which includes *L. crispatus, L. iners, L. jensenii*, and *L. gasseri* (Łaniewski et al., 2018; Kovachev, 2020). A previous study suggested that bacterial community state types dominated by *L. gasseri* might be related with the fastest clearance of acute HPV infection (Brotman et al., 2014). Unlike *L. crispatus, L. iners* produces small amounts of lactic acid without the production of reported host-protective peptide. Cervicovaginal microbiota of transiently HPV-infected women is dominated by *L. iners* (Qingqing et al., 2021), probably because *L. iners* are able to adapt to various pH environments and apparently lack genes for the synthesis of bacteriocin, all of which creates conditions for abnormal cervicovaginal bacteria to proliferate (Macklaim et al., 2011; Mitra et al., 2016). The predominant microbiota in vaginal flora samples was *L. crispatus* or *L. iners*, whereas individuals with a low-*Lactobacillus* vaginal microbiota usually have the colonizedzation of bacteria such as *Gardnerella, Prevotella*, and *Sneathia* (Ravel et al., 2011; Callahan et al., 2017; Serrano et al., 2019). In our study, only 28.89% of patients cleared HPV following treatment with IFN plus vaginal *Lactobacillus* spp. Higher levels of non- *Lactobacillus* dominant bacteria, including *S. amnii, E. faecalis, S. haemolyticus, S. intermedius*, *P. amnii*, and *P. timonensis*, were found in the HPV-persistent group than those in the HPV-cleared group, indicating that women with a high abundance of these bacteria have more difficulty in clearing HPV (Ritu et al., 2019; Chao et al., 2020).

We further classified patients based on their HPV subtypes and assessed the relationship between HPV phylogenetic groups and the composition of the vaginal flora. Our results indicate that *L. iners* is the primary bacterial species that is connected with HPV subtypes. Some studies have demonstrated a link between greater cervical microbiome (CVM) diversity and prevalence of Hr-HPV infection and/or cervical abnormalities (vs. HPV negative) (Audirac-Chalifour et al., 2016; Dareng et al., 2016). Increased alpha diversity was associated with Hr-HPV positivity that was associated with increasing disease severity (Mitra et al., 2015; Klein et al., 2019). Consistent with the previous study, women with multiple HPV types infection showed higher bacterial diversity, with higher diversity being displayed by women with a single HPV type infection than women with no HPV infection (Cheng et al., 2020). Of note, in the HPV-persistent group, women showed significantly higher bacterial diversity than the HPV-cleared group. Presently, evidence on the relationship between CVM diversity and cervical neoplasia severity is conflicting (Mitra et al., 2015; Seo et al., 2016). Because most studies focusing on the natural course of HPV and the microbiome are cross-sectional, it is difficult to decipher potential causality. The potential mechanisms underlying the potential interactions between HPV and microbiota need to be revealed (Cheng et al., 2020).

Probiotics are believed to exert a helpful influence on a wide range of diseases. Compared with the development of novel anti-inflammatory drugs, it may be less costly to find novel approaches, i.e., probiotics that can change the vaginal flora environment by playing a direct role in vaginal flora (Nami et al., 2014; Nami et al., 2014; Eslami et al., 2016; Wang et al., 2018). Currently, probiotic strains of *lactobacillus* administered vaginally by suppository or vaginal ovule have been explored (Knackstedt et al., 2020). Our clinical experience shows that probiotics, as adjunctive therapy for interferon, have a good therapeutic effect on HPV clearance, but also have some efficacy in the clearance of chlamydia and mycoplasma (data not shown). Thus, it is crucial to provide a completely new method to cure diseases by monitoring specific bacterium associated with HPV infection and controlling vaginal flora (Li et al., 2020).

## Data availability statement

The data presented in the study are deposited in the NCBI repository, accession number PRJNA913296.

## Ethics statement

The studies involving human participants were reviewed and approved by The Research Ethics Boards at the First Affiliated Hospital of Shantou University Medical College (No. 201561). Written informed consent for participation was not required for this study in accordance with the national legislation and the institutional requirements.

## Author contributions

JC conceived the study and designed the experiments. MZ and XL performed experiments and analyzed data. XJ and JC wrote the manuscript. FY, SX, XH, and XC revised the manuscript extensively. All of the authors have discussed and approved the final version of the manuscript.

## Supplementary material

Supplementary Figure 1 Schematic diagram of the experimental design from the sample collection to statistic alanalyses.

## References

[B1] Anahtar M. N. Byrne E. H. Doherty K. E. Bowman B. A. Yamamoto H. S. Soumillon M.. (2015). Cervicovaginal bacteria are a major modulator of host inflammatory responses in the female genital tract. Immunity. 42 (5), 965–976. doi: 10.1016/j.immuni.2015.04.019 25992865 PMC4461369

[B2] Audirac-Chalifour A. Torres-Poveda K. Bahena-Román M. Téllez-Sosa J. Martínez-Barnetche J. Cortina-Ceballos B.. (2016). Cervical microbiome and cytokine profile at various stages of cervical cancer: A pilot study. PLos One 11 (4), e0153274. doi: 10.1371/journal.pone.0153274 27115350 PMC4846060

[B3] Berggrund M. Gustavsson I. Aarnio R. Lindberg J. H. Sanner K. Wikström I.. (2020). Temporal changes in the vaginal microbiota in self-samples and its association with persistent HPV16 infection and CIN2. Virol. J. 17 (1), 147. doi: 10.1186/s12985-020-01420-z 33028395 PMC7541248

[B4] Borgdorff H. Gautam R. Armstrong S. D. Xia D. Ndayisaba G. F. van Teijlingen N. H.. (2016). Cervicovaginal microbiome dysbiosis is associated with proteome changes related to alterations of the cervicovaginal mucosal barrier. Mucosal Immunol. 9 (3), 621–633. doi: 10.1038/mi.2015.86 26349657

[B5] Borgogna J. C. Shardell M. D. Santori E. K. Nelson T. M. Rath J. M. Glover E. D.. (2020). The vaginal metabolome and microbiota of cervical HPV-positive and HPV-negative women: a cross-sectional analysis. BJOG. 127 (2), 182–192. doi: 10.1111/1471-0528.15981 31749298 PMC6982399

[B6] Breshears L. M. Edwards V. L. Ravel J. Peterson M. L. (2015). Lactobacillus crispatus inhibits growth of gardnerella vaginalis and neisseria gonorrhoeae on a porcine vaginal mucosa model. BMC Microbiol. 15, 276. doi: 10.1186/s12866-015-0608-0 26652855 PMC4675025

[B7] Brotman R. M. Shardell M. D. Gajer P. Tracy J. K. Zenilman J. M. Ravel J.. (2014). Interplay between the temporal dynamics of the vaginal microbiota and human papillomavirus detection. J. Infect. dis 210 (11), 1723–1733. doi: 10.1093/infdis/jiu330 24943724 PMC4296189

[B8] Brusselaers N. Shrestha S. van de Wijgert J. Verstraelen H. (2019). Vaginal dysbiosis and the risk of human papillomavirus and cervical cancer: systematic review and meta-analysis. Am. J. obstetrics gynecol 221 (1), 9–18.e8. doi: 10.1016/j.ajog.2018.12.011 30550767

[B9] Bychkovsky B. L. Ferreyra M. E. Strasser-Weippl K. Herold C. I. de Lima Lopes G. Jr. Dizon D. S.. (2016). Cervical cancer control in Latin America: A call to action. Cancer. 122 (4), 502–514. doi: 10.1002/cncr.29813 26670695

[B10] Bzhalava D. Mühr L. S. Lagheden C. Ekström J. Forslund O. Dillner J.. (2014). Deep sequencing extends the diversity of human papillomaviruses in human skin. Sci. Rep. 4, 5807. doi: 10.1038/srep05807 25055967 PMC4108911

[B11] Callahan B. J. DiGiulio D. B. Goltsman D. S. A. Sun C. L. Costello E. K. Jeganathan P.. (2017). Replication and refinement of a vaginal microbial signature of preterm birth in two racially distinct cohorts of US women. Proc. Natl. Acad. Sci. United States America 114 (37), 9966–9971. doi: 10.1073/pnas.1705899114 PMC5604014 28847941

[B12] Caporaso J. G. Kuczynski J. Stombaugh J. Bittinger K. Bushman F. D. Costello E. K.. (2010). QIIME allows analysis of high-throughput community sequencing data. Nat. Methods 7 (5), 335–336. doi: 10.1038/nmeth.f.303 20383131 PMC3156573

[B13] Casey B. J. Somerville L. H. Gotlib I. H. Ayduk O. Franklin N. T. Askrend M. K.. (2012). Behavioral and neural correlates of delay of gratification 40 years later. Proc. Natl. Acad. Sci. U.S.A. 2011 Vol 108 No. 36:14998-5003 Ann. neurosciences 19 (1), 27–28. doi: 10.5214/ans.0972.7531.180407 PMC4117069 25205959

[B14] Castle P. E. Giuliano A. R. (2003). Chapter 4: Genital tract infections, cervical inflammation, and antioxidant nutrients–assessing their roles as human papillomavirus cofactors. J. Natl. Cancer Inst Monogr. (31), 29–34. doi: 10.1093/oxfordjournals.jncimonographs.a003478 12807942

[B15] Chan C. K. Aimagambetova G. Ukybassova T. Kongrtay K. Azizan A. (2019). Human papillomavirus infection and cervical cancer: Epidemiology, screening, and vaccination-review of current perspectives. J. Oncol. 2019, 3257939. doi: 10.1155/2019/3257939 31687023 PMC6811952

[B16] Chang A. H. Parsonnet J. (2010). Role of bacteria in oncogenesis. Clin. Microbiol. Rev. 23 (4), 837–857. doi: 10.1128/CMR.00012-10 20930075 PMC2952975

[B17] Chao X. Sun T. Wang S. Tan X. Fan Q. Shi H.. (2020). Research of the potential biomarkers in vaginal microbiome for persistent high-risk human papillomavirus infection. Ann. Trans. Med. 8 (4), 100. doi: 10.21037/atm.2019.12.115 PMC7049000 32175393

[B18] Cheng L. Norenhag J. Hu Y. O. O. Brusselaers N. Fransson E. Ährlund-Richter A.. (2020). Vaginal microbiota and human papillomavirus infection among young Swedish women. NPJ biofilms microbiomes 6 (1), 39. doi: 10.1038/s41522-020-00146-8 33046723 PMC7552401

[B19] Cherpes T. L. Hillier S. L. Meyn L. A. Busch J. L. Krohn M. A. (2008). A delicate balance: risk factors for acquisition of bacterial vaginosis include sexual activity, absence of hydrogen peroxide-producing lactobacilli, black race, and positive herpes simplex virus type 2 serology. Sexually transmitted dis 35 (1), 78–83. doi: 10.1097/OLQ.0b013e318156a5d0 17989585

[B20] Clifford G. M. Smith J. S. Plummer M. Muñoz N. Franceschi S. (2003). Human papillomavirus types in invasive cervical cancer worldwide: a meta-analysis. Br. J. cancer 88 (1), 63–73. doi: 10.1038/sj.bjc.6600688 12556961 PMC2376782

[B21] Curty G. Costa R. L. Siqueira J. D. Meyrelles A. I. Machado E. S. Soares E. A.. (2017). Analysis of the cervical microbiome and potential biomarkers from postpartum HIV-positive women displaying cervical intraepithelial lesions. Sci. Rep. 7 (1), 17364. doi: 10.1038/s41598-017-17351-9 29234019 PMC5727204

[B22] Dareng E. O. Ma B. Famooto A. O. Adebamowo S. N. Offiong R. A. Olaniyan O.. (2016). Prevalent high-risk HPV infection and vaginal microbiota in Nigerian women. Epidemiol. infection 144 (1), 123–137. doi: 10.1017/S0950268815000965 PMC4659743 26062721

[B23] DiGiulio D. B. Callahan B. J. McMurdie P. J. Costello E. K. Lyell D. J. Robaczewska A.. (2015). Temporal and spatial variation of the human microbiota during pregnancy. Proc. Natl. Acad. Sci. United States America 112 (35), 11060–11065. doi: 10.1073/pnas.1502875112 PMC4568272 26283357

[B24] Di Paola M. Sani C. Clemente A. M. Iossa A. Perissi E. Castronovo G.. (2017). Characterization of cervico-vaginal microbiota in women developing persistent high-risk human papillomavirus infection. Sci. Rep. 7 (1), 10200. 28860468 10.1038/s41598-017-09842-6 PMC5579045

[B25] Du J. Näsman A. Carlson J. W. Ramqvist T. Dalianis T. (2011). Prevalence of human papillomavirus (HPV) types in cervical cancer 2003-2008 in Stockholm, Sweden, before public HPV vaccination. Acta Oncol. (Stockholm Sweden) 50 (8), 1215–1219. doi: 10.3109/0284186X.2011.584556 21726177

[B26] Edgar R. C. (2013). UPARSE: highly accurate OTU sequences from microbial amplicon reads. Nat. Methods 10 (10), 996–998. doi: 10.1038/nmeth.2604 23955772

[B27] Eslami S. Hadjati J. Motevaseli E. Mirzaei R. Farashi Bonab S. Ansaripour B.. (2016). Lactobacillus crispatus strain SJ-3C-US induces human dendritic cells (DCs) maturation and confers an anti-inflammatory phenotype to DCs. APMIS Acta pathologica microbiologica immunologica Scandinavica 124 (8), 697–710. doi: 10.1111/apm.12556 27245496

[B28] Fernandes J. V. DEMF T. A. DEA J. C. Cobucci RN M. G. VS A. DEA J. M.. (2015). Link between chronic inflammation and human papillomavirus-induced carcinogenesis (Review). Oncol. letters 9 (3), 1015–1026. doi: 10.3892/ol.2015.2884 PMC4315066 25663851

[B29] Gajer P. Brotman R. M. Bai G. Sakamoto J. Schütte U. M. Zhong X.. (2012). Temporal dynamics of the human vaginal microbiota. Sci. Trans. Med. 4 (132), 132ra52. doi: 10.1126/scitranslmed.3003605 PMC3722878 22553250

[B30] Gao W. Weng J. Gao Y. Chen X. (2013). Comparison of the vaginal microbiota diversity of women with and without human papillomavirus infection: a cross-sectional study. BMC Infect. dis 13, 271. doi: 10.1186/1471-2334-13-271 23758857 PMC3684509

[B31] Gillet E. Meys J. F. Verstraelen H. Verhelst R. De Sutter P. Temmerman M.. (2012). Association between bacterial vaginosis and cervical intraepithelial neoplasia: systematic review and meta-analysis. PLos One 7 (10), e45201. doi: 10.1371/journal.pone.0045201 23056195 PMC3462776

[B32] Godoy-Vitorino F. Romaguera J. Zhao C. Vargas-Robles D. Ortiz-Morales G. Vázquez-Sánchez F.. (2018). Cervicovaginal fungi and bacteria associated with cervical intraepithelial neoplasia and high-risk human papillomavirus infections in a Hispanic population. Front. Microbiol. 9, 2533. doi: 10.3389/fmicb.2018.02533 30405584 PMC6208322

[B33] Haas B. J. Gevers D. Earl A. M. Feldgarden M. Ward D. V. Giannoukos G.. (2011). Chimeric 16S rRNA sequence formation and detection in Sanger and 454-pyrosequenced PCR amplicons. Genome Res. 21 (3), 494–504. doi: 10.1101/gr.112730.110 21212162 PMC3044863

[B34] Ilhan Z. E. Łaniewski P. Thomas N. Roe D. J. Chase D. M. Herbst-Kralovetz M. M. (2019). Deciphering the complex interplay between microbiota, HPV, inflammation and cancer through cervicovaginal metabolic profiling. EBioMedicine. 44, 675–690. doi: 10.1016/j.ebiom.2019.04.028 31027917 PMC6604110

[B35] Kabuki T. Saito T. Kawai Y. Uemura J. Itoh T. (1997). Production, purification and characterization of reutericin 6, a bacteriocin with lytic activity produced by lactobacillus reuteri LA6. Int. J. Food Microbiol. 34 (2), 145–156. doi: 10.1016/S0168-1605(96)01180-4 9039561

[B36] Klein C. Gonzalez D. Samwel K. Kahesa C. Mwaiselage J. Aluthge N.. (2019). Relationship between the cervical microbiome, HIV status, and precancerous lesions. mBio 10 (1), e02785-18. doi: 10.1128/mBio.02785-18 PMC6381280 30782659

[B37] Knackstedt R. Knackstedt T. Gatherwright J. (2020). The role of topical probiotics in skin conditions: A systematic review of animal and human studies and implications for future therapies. Exp. Dermatol. 29 (1), 15–21. doi: 10.1111/exd.14032 31494971

[B38] Kovachev S. M. (2020). Cervical cancer and vaginal microbiota changes. Arch. Microbiol. 202 (2), 323–327. doi: 10.1007/s00203-019-01747-4 31659380

[B39] Langille M. G. Zaneveld J. Caporaso J. G. McDonald D. Knights D. Reyes J. A.. (2013). Predictive functional profiling of microbial communities using 16S rRNA marker gene sequences. Nat. Biotechnol. 31 (9), 814–821. doi: 10.1038/nbt.2676 23975157 PMC3819121

[B40] Łaniewski P. Barnes D. Goulder A. Cui H. Roe D. J. Chase D. M.. (2018). Linking cervicovaginal immune signatures, HPV and microbiota composition in cervical carcinogenesis in non-Hispanic and Hispanic women. Sci. Rep. 8 (1), 7593. doi: 10.1038/s41598-018-25879-7 29765068 PMC5954126

[B41] Łaniewski P. Cui H. Roe D. J. Barnes D. Goulder A. Monk B. J.. (2019). Features of the cervicovaginal microenvironment drive cancer biomarker signatures in patients across cervical carcinogenesis. Sci. Rep. 9 (1), 7333. doi: 10.1038/s41598-019-43849-5 31089160 PMC6517407

[B42] Lee J. E. Lee S. Lee H. Song Y. M. Lee K. Han M. J.. (2013). Association of the vaginal microbiota with human papillomavirus infection in a Korean twin cohort. PLos One 8 (5), e63514. doi: 10.1371/journal.pone.0063514 23717441 PMC3661536

[B43] Lehtinen M. Baussano I. Paavonen J. Vänskä S. Dillner J. (2019). Eradication of human papillomavirus and elimination of HPV-related diseases - scientific basis for global public health policies. Expert Rev. Vaccines 18 (2), 153–160. doi: 10.1080/14760584.2019.1568876 30657348

[B44] Lin D. Kouzy R. Abi Jaoude J. Noticewala S. S. Delgado Medrano A. Y. Klopp A. H.. (2020). Microbiome factors in HPV-driven carcinogenesis and cancers. PLos pathogens 16 (6), e1008524. doi: 10.1371/journal.ppat.1008524 32497113 PMC7271998

[B45] Li Y. Yu T. Yan H. Li D. Yu T. Yuan T.. (2020). Vaginal microbiota and HPV infection: Novel mechanistic insights and therapeutic strategies. Infection Drug resistance 13, 1213–1220. doi: 10.2147/IDR.S210615 32431522 PMC7198448

[B46] Macklaim J. M. Clemente J. C. Knight R. Gloor G. B. Reid G. (2015). Changes in vaginal microbiota following antimicrobial and probiotic therapy. Microbial Ecol. Health disease 26, 27799. doi: 10.3402/mehd.v26.27799 PMC4539393 26282697

[B47] Macklaim J. M. Gloor G. B. Anukam K. C. Cribby S. Reid G. (2011). At The crossroads of vaginal health and disease, the genome sequence of lactobacillus iners AB-1. Proc. Natl. Acad. Sci. United States America 108 Suppl 1 (Suppl 1), 4688–4695. doi: 10.1073/pnas.1000086107 PMC3063587 21059957

[B48] Martin M. (2011). Cutadapt removes adapter sequences from high-throughput sequencing reads. EMBnet J. 17, 10–12. doi: 10.14806/ej.17.1.200

[B49] Mastromarino P. Di Pietro M. Schiavoni G. Nardis C. Gentile M. Sessa R. (2014). Effects of vaginal lactobacilli in chlamydia trachomatis infection. Int. J. Med. Microbiol. IJMM. 304 (5-6), 654–661. doi: 10.1016/j.ijmm.2014.04.006 24875405

[B50] Mitra A. MacIntyre D. A. Lee Y. S. Smith A. Marchesi J. R. Lehne B.. (2015). Cervical intraepithelial neoplasia disease progression is associated with increased vaginal microbiome diversity. Sci. Rep. 5, 16865. doi: 10.1038/srep16865 26574055 PMC4648063

[B51] Mitra A. MacIntyre D. A. Marchesi J. R. Lee Y. S. Bennett P. R. Kyrgiou M. (2016). The vaginal microbiota, human papillomavirus infection and cervical intraepithelial neoplasia: what do we know and where are we going next? Microbiome 4 (1), 58. doi: 10.1186/s40168-016-0203-0 27802830 PMC5088670

[B52] Mitra A. MacIntyre D. A. Ntritsos G. Smith A. Tsilidis K. K. Marchesi J. R.. (2020). The vaginal microbiota associates with the regression of untreated cervical intraepithelial neoplasia 2 lesions. Nat. Commun. 11 (1), 1999. doi: 10.1038/s41467-020-15856-y 32332850 PMC7181700

[B53] Moosa Y. Kwon D. de Oliveira T. Wong E. B. (2020). Determinants of vaginal microbiota composition. Front. Cell. infection Microbiol. 10, 467. doi: 10.3389/fcimb.2020.00467 PMC7492712 32984081

[B54] Nami Y. Abdullah N. Haghshenas B. Radiah D. Rosli R. Khosroushahi A. Y. (2014). Assessment of probiotic potential and anticancer activity of newly isolated vaginal bacterium lactobacillus plantarum 5BL. Microbiol. Immunol. 58 (9), 492–502. doi: 10.1111/1348-0421.12175 25039934

[B55] Nami Y. Abdullah N. Haghshenas B. Radiah D. Rosli R. Yari Khosroushahi A. (2014). A newly isolated probiotic enterococcus faecalis strain from vagina microbiota enhances apoptosis of human cancer cells. J. Appl. Microbiol. 117 (2), 498–508. doi: 10.1111/jam.12531 24775273

[B56] Nieves-Ramírez M. E. Partida-Rodríguez O. Moran P. Serrano-Vázquez A. Pérez-Juárez H. Pérez-Rodríguez M. E.. (2021). Cervical squamous intraepithelial lesions are associated with differences in the vaginal microbiota of Mexican women. Microbiol. spectrum 9 (2), e0014321. doi: 10.1128/Spectrum.00143-21 PMC8515943 34643408

[B57] Norenhag J. Du J. Olovsson M. Verstraelen H. Engstrand L. Brusselaers N. (2020). The vaginal microbiota, human papillomavirus and cervical dysplasia: a systematic review and network meta-analysis. BJOG an Int. J. obstetrics gynaecol 127 (2), 171–180. doi: 10.1111/1471-0528.15854 31237400

[B58] Oliveira G. H. Schirger A. (2003). Images in clinical medicine. page kidney. New Engl. J. Med. 348 (2), 129. doi: 10.1056/NEJMicm020037 12519923

[B59] Palma E. Recine N. Domenici L. Giorgini M. Pierangeli A. Panici P. B. (2018). Long-term lactobacillus rhamnosus BMX 54 application to restore a balanced vaginal ecosystem: a promising solution against HPV-infection. BMC Infect. dis 18 (1), 13. doi: 10.1186/s12879-017-2938-z 29304768 PMC5756375

[B60] Petrova M. I. van den Broek M. Balzarini J. Vanderleyden J. Lebeer S. (2013). Vaginal microbiota and its role in HIV transmission and infection. FEMS Microbiol. Rev. 37 (5), 762–792. doi: 10.1111/1574-6976.12029 23789590

[B61] Piyathilake C. J. Ollberding N. J. Kumar R. Macaluso M. Alvarez R. D. Morrow C. D. (2016). Cervical microbiota associated with higher grade cervical intraepithelial neoplasia in women infected with high-risk human papillomaviruses. Cancer Prev. Res. (Philadelphia Pa). 9 (5), 357–366. doi: 10.1158/1940-6207.CAPR-15-0350 PMC4869983 26935422

[B62] Pyeon D. Pearce S. M. Lank S. M. Ahlquist P. Lambert P. F. (2009). Establishment of human papillomavirus infection requires cell cycle progression. PLos pathogens 5 (2), e1000318. doi: 10.1371/journal.ppat.1000318 19247434 PMC2642596

[B63] Qingqing B. Jie Z. Songben Q. Juan C. Lei Z. Mu X. (2021). Cervicovaginal microbiota dysbiosis correlates with HPV persistent infection. Microbial pathogenesis 152, 104617. doi: 10.1016/j.micpath.2020.104617 33207260

[B64] Quast C. Pruesse E. Yilmaz P. Gerken J. Schweer T. Yarza P.. (2013). The SILVA ribosomal RNA gene database project: improved data processing and web-based tools. Nucleic Acids Res. 41, D590–D596. doi: 10.1093/nar/gks1219 23193283 PMC3531112

[B65] Ravel J. Gajer P. Abdo Z. Schneider G. M. Koenig S. S. McCulle S. L.. (2011). Vaginal microbiome of reproductive-age women. Proc. Natl. Acad. Sci. United States America 108 Suppl 1 (Suppl 1), 4680–4687. doi: 10.1073/pnas.1002611107 PMC3063603 20534435

[B66] Ritu W. Enqi W. Zheng S. Wang J. Ling Y. Wang Y. (2019). Evaluation of the associations between cervical microbiota and HPV infection, clearance, and persistence in cytologically normal women. Cancer Prev. Res. (Philadelphia Pa). 12 (1), 43–56. doi: 10.1158/1940-6207.CAPR-18-0233 30463989

[B67] Rognes T. Flouri T. Nichols B. Quince C. Mahé F. (2016). VSEARCH: a versatile open source tool for metagenomics. PeerJ. 4, e2584. doi: 10.7717/peerj.2584 27781170 PMC5075697

[B68] Romero R. Hassan S. S. Gajer P. Tarca A. L. Fadrosh D. W. Nikita L.. (2014). The composition and stability of the vaginal microbiota of normal pregnant women is different from that of non-pregnant women. Microbiome. 2 (1), 4. doi: 10.1186/2049-2618-2-4 24484853 PMC3916806

[B69] Romero-Morelos P. Bandala C. Jiménez-Tenorio J. Valdespino-Zavala M. Rodríguez-Esquivel M. Gama-Ríos R. A.. (2019). Vaginosis-associated bacteria and its association with HPV infection. Med clinica 152 (1), 1–5. doi: 10.1016/j.medcle.2018.11.005 29544661

[B70] Schiffman M. Doorbar J. Wentzensen N. de Sanjosé S. Fakhry C. Monk B. J.. (2016). Carcinogenic human papillomavirus infection. Nat. Rev. Dis. primers 2, 16086. doi: 10.1038/nrdp.2016.86 27905473

[B71] Seo S. S. Oh H. Y. Lee J. K. Kong J. S. Lee D. O. Kim M. K. (2016). Combined effect of diet and cervical microbiome on the risk of cervical intraepithelial neoplasia. Clin. Nutr. (Edinburgh Scotland) 35 (6), 1434–1441. doi: 10.1016/j.clnu.2016.03.019 27075319

[B72] Serrano M. G. Parikh H. I. Brooks J. P. Edwards D. J. Arodz T. J. Edupuganti L.. (2019). Racioethnic diversity in the dynamics of the vaginal microbiome during pregnancy. Nat. Med. 25 (6), 1001–1011. doi: 10.1038/s41591-019-0465-8 31142850 PMC6746180

[B73] Shannon B. Yi T. J. Perusini S. Gajer P. Ma B. Humphrys M. S.. (2017). Association of HPV infection and clearance with cervicovaginal immunology and the vaginal microbiota. Mucosal Immunol. 10 (5), 1310–1319. doi: 10.1038/mi.2016.129 28120845 PMC5526752

[B74] Srinivasan S. Liu C. Mitchell C. M. Fiedler T. L. Thomas K. K. Agnew K. J.. (2010). Temporal variability of human vaginal bacteria and relationship with bacterial vaginosis. PLos One 5 (4), e10197. doi: 10.1371/journal.pone.0010197 20419168 PMC2855365

[B75] Stoyancheva G. Marzotto M. Dellaglio F. Torriani S. (2014). Bacteriocin production and gene sequencing analysis from vaginal lactobacillus strains. Arch. Microbiol. 196 (9), 645–653. doi: 10.1007/s00203-014-1003-1 24919535

[B76] Usyk M. Zolnik C. P. Castle P. E. Porras C. Herrero R. Gradissimo A.. (2020). Cervicovaginal microbiome and natural history of HPV in a longitudinal study. PLos pathogens 16 (3), e1008376. doi: 10.1371/journal.ppat.1008376 32214382 PMC7098574

[B77] Verhoeven V. Renard N. Makar A. Van Royen P. Bogers J. P. Lardon F.. (2013). Probiotics enhance the clearance of human papillomavirus-related cervical lesions: a prospective controlled pilot study. Eur. J. Cancer Prev. Off. J. Eur. Cancer Prev. Organisation (ECP). 22 (1), 46–51. doi: 10.1097/CEJ.0b013e328355ed23 22706167

[B78] Walboomers J. M. Jacobs M. V. Manos M. M. Bosch F. X. Kummer J. A. Shah K. V.. (1999). Human papillomavirus is a necessary cause of invasive cervical cancer worldwide. J. pathology 189 (1), 12–19. doi: 10.1002/(SICI)1096-9896(199909)189:1<12::AID-PATH431>3.0.CO;2-F 10451482

[B79] Wang Q. Garrity G. M. Tiedje J. M. Cole J. R. (2007). Naive Bayesian classifier for rapid assignment of rRNA sequences into the new bacterial taxonomy. Appl. Environ. Microbiol. 73 (16), 5261–5267. doi: 10.1128/AEM.00062-07 17586664 PMC1950982

[B80] Wang K. D. Xu D. J. Wang B. Y. Yan D. H. Lv Z. Su J. R. (2018). Inhibitory effect of vaginal lactobacillus supernatants on cervical cancer cells. Probiotics antimicrobial proteins 10 (2), 236–242. doi: 10.1007/s12602-017-9339-x 29071554

[B81] Wilkinson E. J. Cox J. T. Selim M. A. O'Connor D. M. (2015). Evolution of terminology for human-papillomavirus-infection-related vulvar squamous intraepithelial lesions. J. lower genital tract disease 19 (1), 81–87. doi: 10.1097/LGT.0000000000000049 24832173

