# Supplementary figures and images for: Roles of vaginal flora in human papillomavirus infection, virus persistence and clearance
Source: Front Cell Infect Microbiol. 2023 Jan 4;12:1036869. doi: 10.3389/fcimb.2022.1036869 (PMC9848591; doi:10.3389/fcimb.2022.1036869)

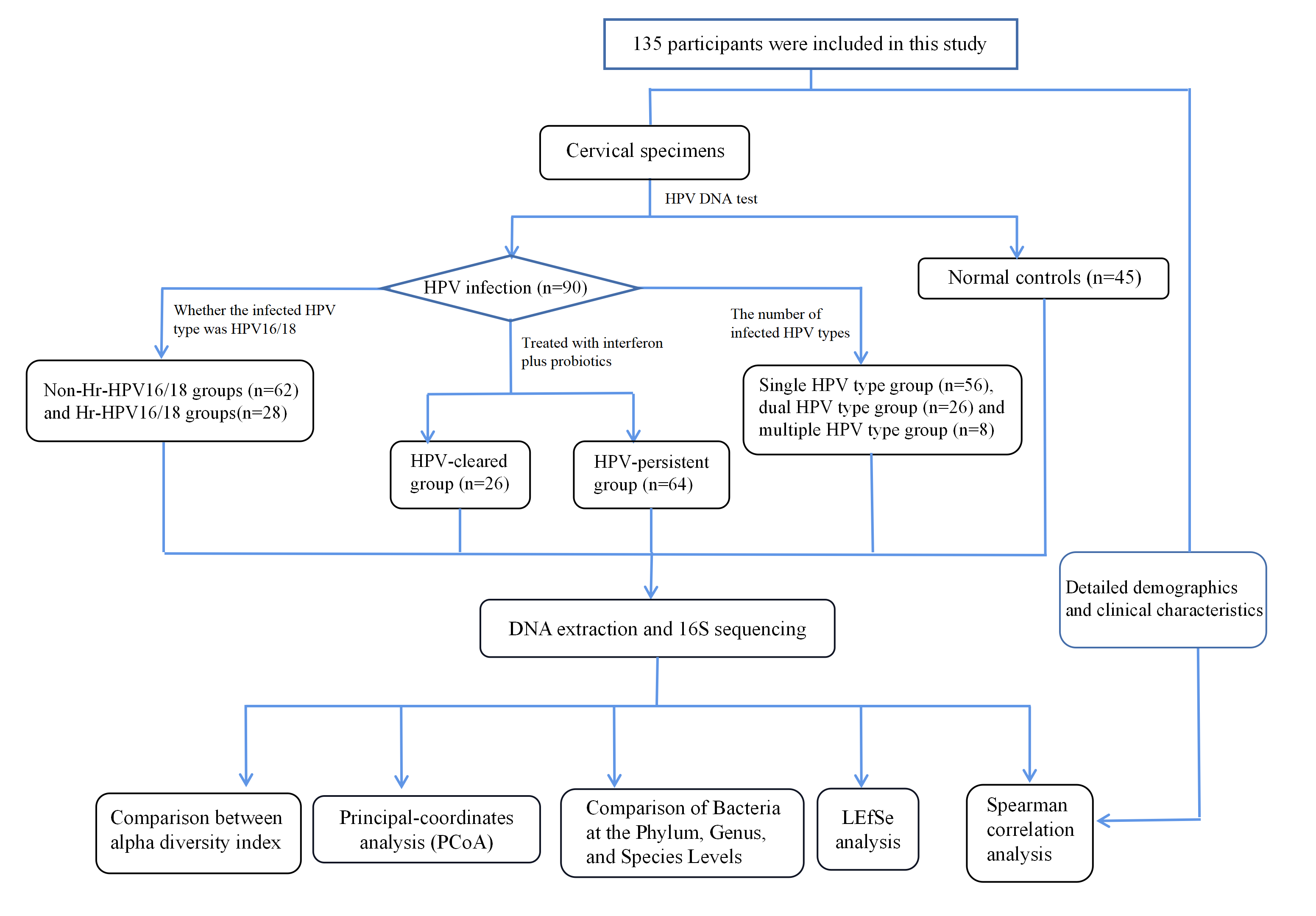

Supplement: Supplementary Figure 1 — Schematic diagram of the experimental design from the sample collection to statistic alanalyses. [file Image_1.tif]
